# The “bad” cholesterol can predict abnormal apolipoprotein B levels in a large unselected outpatient cohort

**DOI:** 10.18632/oncotarget.23759

**Published:** 2017-12-29

**Authors:** Guo-Ming Zhang, Hemant Goyal, Gao-Ming Zhang, Xiao-Bo Ma, Ye-Ting Zhou

**Affiliations:** ^1^ Shuyang People's Hospital, Affiliated Shuyang Hospital, Xuzhou Medical University, Shuyang 223600, China; ^2^ Department of Internal Medicine, Mercer University School of Medicine, Macon 31201, GA, USA

**Keywords:** total cholesterol, high-density lipoprotein cholesterol, low-density lipoprotein cholesterol, apolipoprotein B, reflex test

## Abstract

**Background:**

The significant association between total cholesterol (TC), non-high-density lipoprotein cholesterol (non-HDL), and low-density lipoprotein cholesterol (LDL) has been shown to be associated with Apolipoprotein B (Apo B). The objective of this study was to assess whether abnormal levels of TC, non-HDL and LDL can be used as predictors of abnormal serum Apo B levels.

**Results:**

TC (*r* = 0.752), non-HDL (*r* = 0.799), and LDL(*r* = 0.817) were significantly positively correlated with Apo B. Areas under the curve of TC, non-HDL, and LDL for predicting abnormal Apo B (>1.10 g/L) were 0.906, 0.918, and 0.928, respectively. The optimal thresholds of prediction of abnormal Apo B were 5.13 mmol/L for TC, 4.23 mmol/L for non-HDL, and 3.34 mmol/L for LDL. At these optimal thresholds of TC, non-HDL and LDL, less than 1.13%, 1.67%, and 0.62% of tests with abnormal Apo B results would have been missed, but approximately 69.4%, 79.7%, and 73.2% of the performed Apo B tests could have been eliminated, respectively.

**Conclusions:**

Apo B levels of unselected outpatients need be not tested (especially when LDL < 3.34 mmol/L, non-HDL < 4.23 mmol/L, and/or TC < 5.13 mmol/L). It will result in 69% reduction in number of ordered Apo B tests. LDL was significantly better than the TC and non-HDL for predicting abnormal Apo B indicating that Apo B needn't tested when LDL level is normal.

**Methods:**

We retrospectively analyzed results of TC, HDL, LDL, and Apo B in a large cohort of unselected outpatients (*n* = 5486) in Shuyang People's Hospital, Shuyang, China. Non-HDL was calculated by deducting HDL from TC. Correlations between TC, non-HDL, LDL, and Apo B were analyzed by using Spearman's rho approach. Receiver operating characteristics curve analysis was used to evaluate the predictive value of TC, non-HDL, and LDL for abnormal Apo B.

## INTRODUCTION

Apolipoprotein B (Apo B) levels are generally tested for patients with cardiac diseases and/or hyperlipidemia. This test is ordered for the diagnosis of dyslipidemia, especially when someone has abnormally elevated cholesterol and triglyceride levels [1]. Total cholesterol (TC), high-density lipoprotein cholesterol (HDL), and low-density lipoprotein cholesterol (LDL) are widely used as a part of the lipid panel in clinical practice [2]. The cholesterol and Apo B are very important because they are a marker of risk of development of coronary artery disease and their treatment can significantly reduce the morbidity and mortality in patients with established cardiovascular disorders [1, 3]. The“bad” cholesterol include TC, LDL, and non-HDL (non-HDL=TC-HDL) [4]. In China, usually serum cholesterol and Apo B levels are ordered together for evaluation for lipid derangement. Since the Apo B levels are used to mirror the concentrations of TC, LDL, and non-HDL, and levels of Apo B tend to follow along with cholesterol (TC, LDL, and non-HDL) and these cholesterols are highly correlated with each other. We hypothesized that elevated cholesterol could be used as an indirect measure of Apo B concentration. In this study, we analyze whether measurement of cholesterol level can limit ordering of Apo B tests in the clinical practice.

## RESULTS

**Figure 1:**
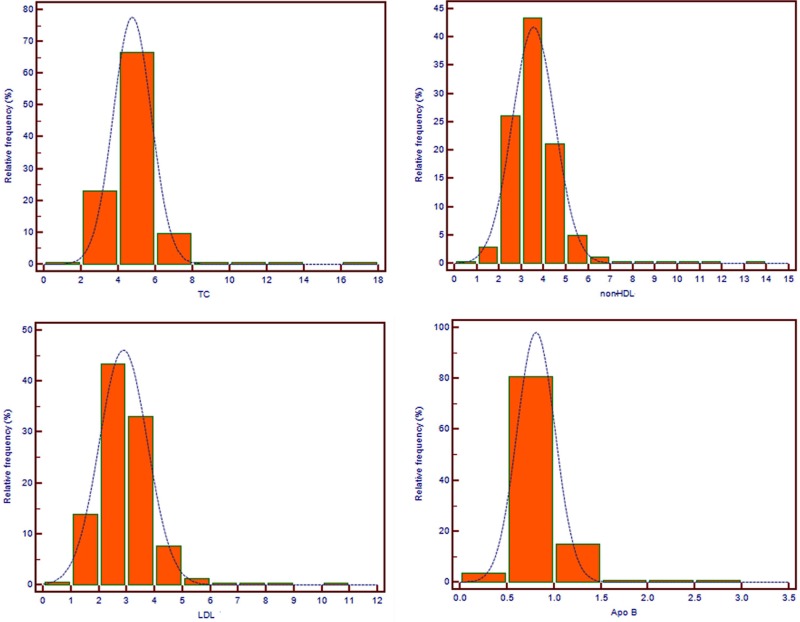
Histograms of cholesterol and Apolipoprotein B TC: Total cholesterol; LDL: low-density lipoprotein cholesterol; non-HDL: non-high-density lipoprotein cholesterol; Apo B: ApolipoproteinB.

In total, 5486 consecutive subjects included in the analysis who underwent testing for TC, HDL, LDL, and Apo B between October 1, 2013, and April 1, 2017. Baseline results of these tests are tabulated in Table 1. TC, HDL, and non-HDL of the male study population were lower than females (*p* < 0.001). Figure 1 shows histograms depicting TC, non-HDL, LDL, and Apo B distributions among the study subjects.

**Table 1:** The characteristics of the participants

Parameters	Female	Male	P^*^
	2439	3047	
Total Cholesterol (mmol/L)	4.76 (4.13–5.46)	4.57 (3.99–5.20)	< 0.001
LDL (mmol/L)	2.82 (2.26–3.43)	2.85 (2.29–3.36)	0.919
non-HDL (mmol/L)	3.48 (2.92–4.17)	3.44 (2.90–4.01)	0.006
HDL (mmol/L)	1.23 (1.04–1.45)	1.10 (0.96–1.28)	< 0.001
Apolipoprotein B (g/L)	0.79 (0.66–0.93)	0.79 (0.67–0.91)	0.402

All data is represented by median and interquartile range.

^*^Kruskal - Wallis test was used to determine a significant gender group.

LDL: low-density lipoprotein cholesterol; HDL: high-density lipoprotein cholesterol; non-HDL: non-high-density lipoprotein cholesterol.

### Correlation between TC, non-HDL, LDL and Apo B

**Figure 2:**
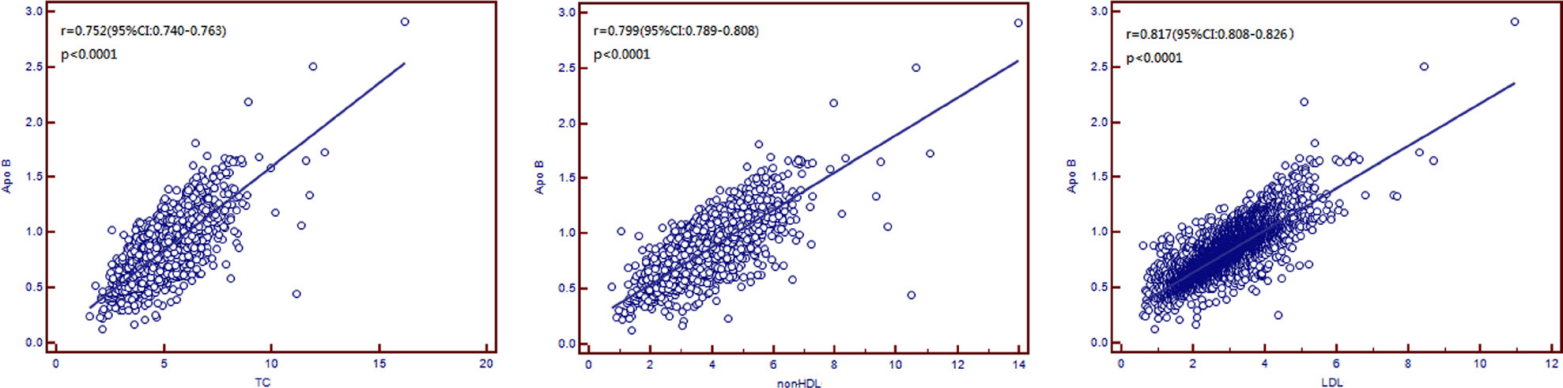
Scatter plots for Apolipoprotein B (Apo B) and total cholesterol (TC), non-density lipoprotein cholesterol (non-HDL), and low-density lipoprotein cholesterol (LDL)

As shown in Figure 2, TC (*r* = 0.752, 95% Confidence Interval, CI: 0.740–0.763, *p* < 0.0001), non-HDL (*r* = 0.799, 95% CI: 0.789–0.808, *p* < 0.0001), and LDL (*r* = 0.817, 95% CI: 0.808–0.826, *p* < 0.0001) significantly correlated with levels of Apo B.

### Accuracy of TC, non-HDL, and LDL levels in predicting abnormal Apo B levels

**Figure 3:**
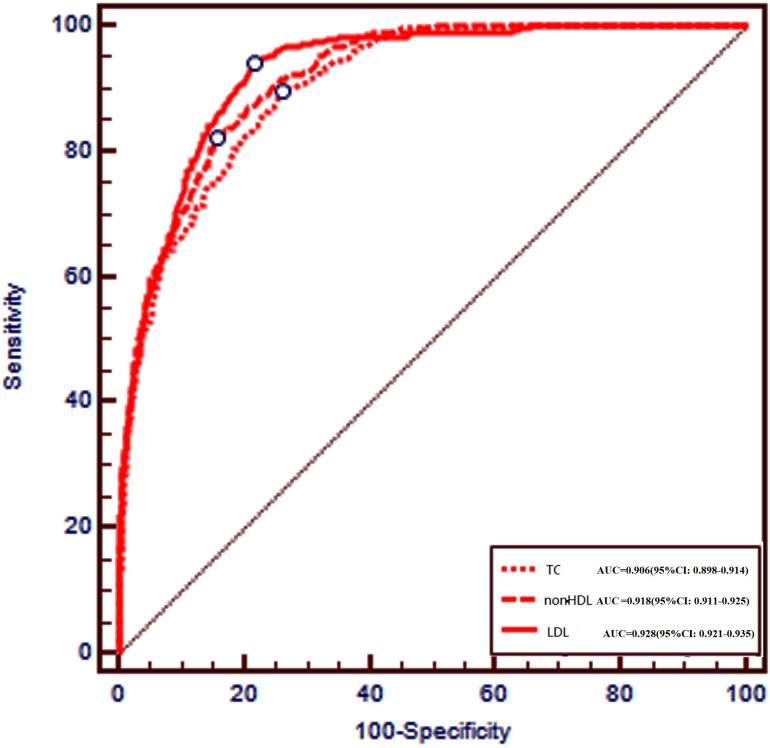
Receiver operating characteristics curves of total cholesterol (TC), non -density lipoprotein cholesterol (non-HDL), and low-density lipoprotein cholesterol (LDL) for predicting abnormal Apolipoprotein B (Apo B)

Figure 3 shows the ROC curves of TC, non-HDL, and LDL for predicting abnormal Apo B levels. Table 2 shows their area under curves (AUC), which were 0.906, 0.918, and 0.928 for TC, non-HDL, and LDL, respectively. Nevertheless, the threshold levels of TC, non-HDL, and LDL for predicting abnormal Apo B were 5.13 mmol/L, 4.23 mmol/L, and 3.34 mmol/L, respectively. The diagnostic performance of LDL in predicting abnormal Apo B levels was notably better than that of the TC and non-HDL. At these threshold levels of TC, non-HDL and LDL, only 1.13%, 1.67%, and 0.62% of tests with abnormal Apo B might have been missed, but about 69.4%, 79.7%, and 73.2% of Apo B test orders would have been avoided, respectively.

**Table 2:** The optimal threshold and accuracy of Total Cholesterol (TC), non-high-density lipoprotein cholesterol (non-HDL), and low-density lipoprotein cholesterol (LDL), and its performance in predicting abnormal Apolipoprotein B(Apo B)

	Apo B = 1.10 g/L		
	TC	non-HDL	LDL
AUC	0.906 95% CI:0.898–0.914	0.918 95% CI:0.911–0.925	0.928 95% CI:0.921–0.935
Thresholds	5.13 mmol/L	4.23 mmol/L	3.34 mmol/L
Sensitivity	89.3 95% CI:85.8–92.1	81.8 95% CI:77.7–85.5	93.8 95% CI:90.9–95.9
Specificity	74.0 95% CI:72.8–75.2	84.6 95% CI:83.6-85.6	78.5 95% CI:77.3–79.6
Not test	69.4% (3806/5486)	79.7% (4374/5486)	73.2% (4016/5486)
Missing test	1.13% (43/3806)	1.67% (73/4374)	0.62% (25/4016)
*Z* value	5.442	4.215	2.108
*P*-value	<0.0001^a^	<0.0001^b^	0.035^c^

CI: Confidence Interval; ^a^Compare with non-HDL groups; ^b^Compare with LDL groups; ^c^Compare with non-HDL groups.

## DISCUSSION

In this study, we retrospectively analyzed results of TC, HDL, LDL, and Apo B and their interrelationship to predict the abnormal Apo B levels in a large cohort of unselected outpatients who consecutively visited Shuyang People's Hospital for testing of lipid panel. Furthermore, we found that levels of TC, non-HDL and LDL positively correlated with levels of Apo B test. In other words, subjects with high TC and high non-HDL also had high LDL levels and vice versa. Moreover, the concentrations of Apo B tend to increase or decrease along with LDL level. Therefore, we propose that for subjects with high TC, non-HDL and LDL, the level of Apo B need not be tested which is supported by the results of ROC of TC, non-HDL, and LDL for predicting elevated Apo B levels. The AUC of TC, non-HDL and LDL was more than 0.90, indicating that these tests have good accuracy for predicting elevated Apo B levels.

As we all know, the combination of two biomarkers for diagnostic test will raise the sensitivity, and drop the specificity. If the combination of two biomarkers for predicting abnormal Apo B levels is used, the missing test rate will increase in this study. In the current study, it is implied that LDL was significantly a better test for predicting abnormal Apo B than that of TC and non-HDL.

The optimal threshold levels of TC, non-HDL and LDL for predicting abnormal Apo B levels were 5.13 mmol/L, 4.23 mmol/L, and 3.34 mmol/L respectively. At these thresholds, less than 1.13%, 1.67%, and 0.62% of abnormal Apo B results would have been missed, however, the need of approximately 69.4%, 79.7%, and 73.2% of Apo B tests could be reduced, respectively. The cost of one Apo B test is approximately 25RMB ($4 USD) in China, as per the data from Chinese Medical Care Database [5]. If abnormal LDL was used as a surrogate for abnormal Apo B, approximately 220 million Apo B tests would have been avoided, with a saving of about 880 million USD annually!

This study has some limitations too. First, the study results come from a large but single center, and data collection is retrospective. Second, the results of this study can only be applied to outpatient population and further studies should be performed to verify the usefulness of our results in inpatients. Three, this study is a retrospective study, and all data was from Our Department of Laboratory Medicine.

The results of our study suggest that need for orders of Apo B tests could have been eliminated if TC, non-HDL, and LDL are used as a marker for abnormal Apo B levels. This approach could save millions of healthcare dollars. Apo B levels of unselected outpatients need be tested only when elevated TC or elevated non-HDL or elevated LDL.

## MATERIALS AND METHODS

### Study cohort and data extraction

We retrospectively analyzed the results of TC, HDL, LDL and Apo B levels which were ordered for the patients in Shuyang People's Hospital in outpatient setting from October 2013 to April 2017. These results were measured using TBA2000FR biochemical analyzer (Toshiba Co., Ltd., Japan) and were available in hospital's laboratory information system (LIS). The Liquid Double-reagent kit for measuring TC (COD-PAP Method), HDL (Direct Clearance Method), LDL(Direct Clearance Method) and Apo B (immunoturbidimetry) are from KEHUA Biosciences, Shanghai, China. The quality control of these tests is checked and monitored every day in the department of laboratory medicine of Shuyang People's Hospital. The external quality assessment scheme of these tests was performed twice a year in Jiangsu center for clinical laboratories to validate the quality results. The ethics committee of Shuyang People's Hospital approved this study in 2017.

### Statistical analysis and calculation

Non-HDL levels were calculated by deducting HDL from TC levels [4]. The relationship was analyzed using Spearman's approach. Receiver operating characteristics (ROC) curve analysis was used to evaluate the predictive accuracy of TC, non-HDL, and LDL (1 mg/dL × 0.02586 = 0.02586 mmol/L) [6, 7] for abnormal Apo B testing. The reference value of Apo B is ≤1.10 g/L in our Department of Laboratory Medicine. Therefore, the abnormal Apo B level was considered as >1.10 g/L. The test results were gathered on Microsoft Excel*^®^* Sheet and statistical analyses were performed using and MedCalc 15.2.2 (MedCalc Software, Ostend, Belgium). A *p*-value of less than 0.05 was considered as statistically significant.
